# Ethnic Similarities and Differences in the Relationship between Beta Cell Mass and Diabetes

**DOI:** 10.3390/jcm6120113

**Published:** 2017-11-30

**Authors:** Jun Inaishi, Yoshifumi Saisho

**Affiliations:** Department of Internal Medicine, Keio University School of Medicine, 35 Shinanomachi, Shinjuku-ku, Tokyo 160-8582, Japan; inaina1714jun9@yahoo.co.jp

**Keywords:** beta cell mass, diabetes, obesity, human pancreas, Japanese

## Abstract

Recent evidence has revealed that a change of functional beta cell mass is an essential factor of the pathophysiology of type 2 diabetes (T2DM). Since beta cell dysfunction is not only present in T2DM but also progressively worsens with disease duration, to preserve or recover functional beta cell mass is important in both prevention of the development of T2DM and therapeutic strategies for T2DM. Furthermore, ethnic difference in functional beta cell mass may also need to be taken into account. Recent evidences suggest that Asians have less beta cell functional capacity compared with Caucasians. Preservation or recovery of functional beta cell mass seems to be further emphasized for Asians because of the limited capacity of beta cell. This review summarizes the current knowledge on beta cell dysfunction in T2DM and discusses the similarities and differences in functional beta cell mass between ethnicities in the face of obesity and T2DM.

## 1. Introduction

The number of diabetic patients is continuing to increase all over the world. Since the incidence of diabetic complications and the cost of treatment remain major issues, diabetes is a serious problem in the world, and optimal prevention and therapy of diabetes are needed as soon as possible. Of people with diabetes, more than 90% have type 2 diabetes (T2DM). Lifestyle factors and ethnicity are known determinants of the development of T2DM [1]. Obesity is a major risk factor for the development of T2DM [2].

However, Asians can develop T2DM even if they are not obese [3]. Although T2DM in Asians is also characterized by obesity and insulin resistance, beta cell function may contribute to the difference in phenotype of T2DM between ethnicities. Recent studies have revealed that a deficit of functional beta cells is a core problem in T2DM [4]. In this review, current knowledge regarding beta cell dysfunction in T2DM is summarized, and similarities and differences in functional beta cell mass between ethnicities in the face of obesity and T2DM are discussed.

## 2. Beta Cell Mass and Function in the Face of Obesity and Diabetes

### 2.1. Change in Beta Cell Function and Beta Cell Mass in T2DM

In patients with T2DM, plasma insulin concentration is often raised. However, the higher plasma insulin concentration demands greater insulin due to decreased insulin sensitivity. Therefore, true beta cell function should be assessed with adjustment for concomitant insulin sensitivity. Although the gold standard for assessment of beta cell function is acute insulin response (AIR) or maximum AIR (AIRmax) [5], it is difficult to perform measurements with the hyperglycemic clamp technique using an artificial pancreas in clinical settings. Hence, there are various measures of beta cell function, as shown in Table 1.

Using the disposition index, beta cell function in patients with impaired glucose tolerance (IGT) is decreased and is even lower in patients with T2DM [6]. Several studies have consistently shown that beta cell function is decreased in patients with T2DM [7,8]. Furthermore, based on histological analysis, Butler et al. and other groups have reported that beta cell mass as well as beta cell function is decreased in patients with T2DM [9]. They have reported an approximately 65% beta cell mass decrease in people with T2DM compared with non-diabetic controls matched for age and BMI. Other groups have also reported that beta cell mass in patients with T2DM is reduced by 30–40% [10,11].

A recent study suggested that a number of mechanisms are associated with the deficit of beta cell in people with T2DM [12]. The amount of beta cell mass is regulated by new beta cell formation and beta cell loss. Beta cell apoptosis has been shown to be increased in patients with T2DM, while both beta cell replication and neogenesis were unchanged in an autopsy study [9], suggesting the main cause of reduced beta cell mass in T2DM is an increase in beta cell loss. Increased workload of beta cells may result in beta cell death through various factors such as hyperglycemia [13], oxidative stress [14], endoplasmic reticulum stress [15], inflammatory cytokines [16], and amyloid deposition [17].

As a mechanism of beta cell loss, transdifferentiation of beta cells to alpha cells has also been suggested. Recent rodent studies have shown dedifferentiation of beta cells to alpha cells [18], although whether an alpha cell mass increase occurs in patients with T2DM remains unclear. Alpha cell mass has been reported to be unchanged in patients with T2DM [19,20], while the others have reported that alpha cell area increased in those with T2DM [12]. In a study using human pancreatic islets, dedifferentiated cells from beta cells increased and the number of hormone-negative cells was 3-fold higher in diabetics compared with non-diabetic controls, suggesting that beta cells become dedifferentiated and convert to alpha- or delta-like cells in patients with T2DM [21]. This report has also been suggested that the underlying basis of the beta cell deficit in T2DM is transdifferentiation rather than beta-cell loss through apoptosis. Butler et al. reported the presence of endocrine cells with altered cell identity in patients with T2DM, but this process did not account for the deficit of beta cells in T2DM since these cells were very few [22].

Thus, the mechanisms of beta cell deficit in humans with T2DM remain unclear. Nonetheless, beta cell mass progressively declines with disease duration, and it has actually been reported that beta cell mass is negatively correlated with the duration of T2DM [11].

### 2.2. Change in Beta Cell Mass with Obesity

The physiological changes in beta cell mass during the development of T2DM remain less clear. The plasma insulin level in obese subjects is increased approximately 2- to 3-fold to compensate insulin resistance [23], and an increase in beta cell mass with obesity is assumed. In a histological analysis, beta cell mass in obese individuals increases by approximately 20–50% compared with controls who were not obese in the Caucasian population without diabetes [24]. An increase in beta cell replication was not observed in obese humans, but it is possible that the increase in replication is too small and gradual to be measured in humans. Although there is a significant difference in beta cell mass between lean and obese humans, not only the source but also the timing of the increased beta cells in obese humans remain unclear.

In the transition period between normoglycemia and T2DM, several reports have reported that beta cell mass in patients with IGT was reduced by approximately 20–40% [9,25]. These results suggest that beta cell mass starts to decline before the development of T2DM.

### 2.3. Association between Beta Cell Mass and Beta Cell Function

It has been reported that there are relationships between beta cell mass and glucose metabolism such as plasma glucose levels at fasting or 120 min after oral ingestion of 75 g glucose [25], glycated hemoglobin (HbA1c) [25,26], and glycated albumin (GA) [26].

It is more complicated to reveal the relationship between beta cell mass and beta cell function. Using surgically resected pancreas samples, Meier et al. reported that beta cell mass was positively correlated with beta cell function, especially postprandial C-peptide level [27]. This result suggests that beta cell function and beta cell mass seem to be correlated with each other, and both seem to decrease during the development of glucose intolerance. In another study, beta cell dysfunction was markedly improved after overnight beta cell rest by somatostatin administration [28].

Taken together, these findings indicate that it is difficult to separate beta cell function and beta cell mass, although on some occasions they can be dissociated. Thus, rather than separate beta cell mass and function, we use the term “functional beta cell mass” to represent beta cell functional capacity.

## 3. Similarities and Differences in Beta Cell Mass between Ethnicities

### 3.1. Ethnic Difference in Pathophysiological Features of T2DM

It has been reported that Asians are less obese than Caucasians [29], suggesting that plasma insulin in Asians is lower than in Caucasians. Kodama et al. reported an ethnic difference in the insulin secretion–insulin sensitivity relationship in meta-analysis [30]. In their study, average body mass index (BMI) values in Africans, Caucasians, and Asians were 24.7, 24.6, and 21.5 kg/m^2^ in people with normoglycemia and 34.0, 35.1, and 24.9 kg/m^2^ in diabetic people, respectively. Furthermore, individuals with T2DM in Asians were characterized by lower insulin secretion and higher insulin sensitivity compared with Caucasians and Africans across glucose tolerance subgroups. 

Since the average BMI values of Asians with T2DM is <25 kg/m^2^, about half of patients with T2DM are not even obese (i.e., BMI ≥25 kg/m^2^, the definition of obesity in Asian countries). In a Japanese cohort study investigating the effects of insulin secretion and insulin sensitivity according to BMI on the incidence of diabetes, individuals with BMI <23.0 kg/m^2^ developed T2DM mainly through not insulin resistance but impaired insulin secretion [31].

Thus, recent evidence suggests a pathophysiological difference in T2DM development between Asians and Caucasians, and Asians might have less beta cell functional capacity compared with Caucasians.

### 3.2. Beta Cell Mass in Asian Population

We have recently assessed the effects of diabetes and obesity on beta cell mass in Japanese individuals who had undergone pancreatic surgery [26]. As a result, no difference in beta cell mass between lean and obese subjects was observed in both patients with and without T2DM, where obesity was defined as BMI of 25 kg/m^2^ or greater. Additionally, there was no significant correlation between beta cell mass and BMI in either those with or without T2DM. 

Our study using autopsy pancreas and another Japanese study also confirmed these findings [32,33], which, however, are inconsistent with findings in the Caucasian population [24]. This difference might have been affected by the lower obesity in Japanese. However, we also reported that there was no significant increase in beta cell mass in Japanese individuals without diabetes who developed glucocorticoid-induced insulin resistance [20].

Considering the similar incidence of T2DM between ethnicities despite a lower degree of obesity in Japanese [34], these findings suggest that beta cell regenerative capacity in Asians may be limited compared with Caucasians, which is probably derived from both genetic and environmental factors. Although genetic factors for T2DM remain less clear, genome-wide association studies have recently detected genetic loci associated with T2DM, most of which are assumed to relate to beta cells [35], also suggesting the important role of beta cells in the pathogenesis of T2DM. It has been also suggested that forkhead box O1 (FOXO1) is related to the pathogenesis of T2DM since activated FOXO1 leads to decrease beta cell proliferation and compensatory ability in islet [36,37]. Molecular differences between ethnicities contributing to beta cell mass remains unclear and further studies are needed to determine genetic and molecular factors regulating beta cell mass in humans.

**Figure 1:**
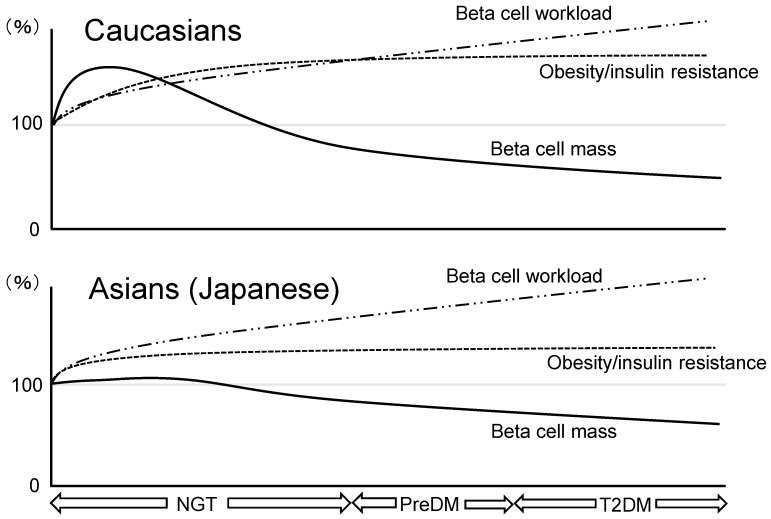
Hypothetical schema of different changes in beta cell mass in relation to obesity/insulin resistance and beta cell workload during the development of glucose intolerance in Caucasians and Asians (Japanese). NGT: normal glucose tolerance; PreDM: prediabetes; T2DM: type 2 diabetes. Beta cell mass increases to adapt to the increased demand in obese nondiabetic individuals in the Caucasian population, while beta cell mass expansion in the face of insulin resistance is extremely limited in Asians. Despite less obesity/insulin resistance, the limited increase in beta cell mass in Asians results in a similar increase in beta cell workload to that in Caucasians. Excess beta cell workload may eventually cause loss of beta cell mass. Once beta cell mass is reduced, beta cell workload will further increase, resulting in a vicious cycle. With progression to prediabetes and overt diabetes, progressive decline of beta cell mass underlies the disease.

A hypothetical schema of ethnic similarities and differences in beta cell mass during the development of T2DM is shown in Figure 1. In Caucasians, since increased insulin demand due to insulin resistance caused by unhealthy lifestyle such as excessive caloric intake, physical inactivity, and overweightness exceeds the magnitude of beta cell mass expansion, beta cell workload increases as a result. In patients who are susceptible to T2DM, increased beta cell workload may lead to beta cell failure and the development of T2DM. Furthermore, both an increase in beta cell workload and a reduction in beta cell mass continue because insulin resistance usually exists continuously.

On the other hand, because of the limited capacity of beta cell regeneration in Asians compared with Caucasian, excess beta cell workload could be induced with lower obesity and may lead to beta cell failure and the development of T2DM.

## 4. Treatment Strategy for T2DM in Asian Population

The current perspective suggests that beta cell failure and a deficit of functional beta cell mass occur far before the onset of T2DM and possibly even before the onset of IGT [6]. This point emphasizes that it is important to preserve or recover functional beta cell mass as a management and treatment strategy for T2DM [38]. Several clinical trials have shown that lifestyle modification and insulin sensitizers that decrease beta cell workload [39,40], but not insulin secretagogues [41], are effective at preventing the development of T2DM. In the Asian population, reducing beta cell workload seems to be the most effective treatment strategy for T2DM.

**Figure 2:**
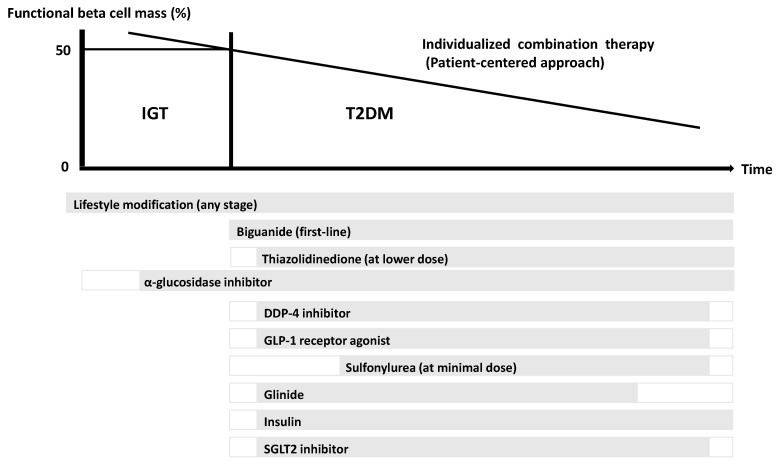
Proposed concept of treatment strategy for type 2 diabetes (T2DM) in Asians in relation to functional beta cell mass. An α-glucosidase inhibitor is partly approved for use in patients with impaired glucose tolerance (IGT) in Japan. Medications not approved in Japan are not included in the figure. Adapted and modified from references 4 and 45 [4,45].

A proposed concept of a treatment strategy for T2DM in Asians in relation to functional beta cell mass is shown in Figure 2. Lifestyle modification such as nutritional therapy, exercise, and weight loss remains the essential factor of treatment of T2DM at any stage of the disease including IGT. Since metformin is effective not only in obese patients but also in lean patients with T2DM [42], and reduces insulin demand by lowering hepatic glucose production, starting therapy by metformin in addition to lifestyle modification should be considered as early a stage of diabetes as possible in patients without contraindication. The use of incretin therapy, which enhances physiological insulin secretion without serious adverse effects, is now widely used for treatment of T2DM. Incretin treatment has been shown to increase beta cell mass in rodents; however, this effect has not been confirmed in humans [43]. On the other hand, insulin secretagogues such as sulphonylureas may be used at only a minimal dose rather to enhance incretin action. Since it has been reported that initial intensive insulin therapy preserves beta cell function thereafter [44], increased risk of hypoglycemia, weight gain, fear of injection, and cost are barriers for this therapy.

Since it is difficult to cure and manage T2DM by only single therapy or agent, an effective combination of current medications in addition to lifestyle modification for the purpose of reduction in beta cell workload is important to preserve or recover functional beta cell mass.

## 5. Conclusions

This review summarized the current knowledge on beta cell dysfunction in T2DM and discussed the similarities and differences in functional beta cell mass between ethnicities in the face of obesity and T2DM.

Considering that Asians, compared with Caucasians, have less beta cell functional capacity, the importance of prevention and therapeutic strategy for T2DM aiming to preserve or recover functional beta cell mass should be emphasized in the Asian population. Few treatment strategies or medications to recover functional beta cell mass have been established yet, and lifestyle modification remains the most fundamental and important therapy for T2DM.

## Figures and Tables

**Table 1:** Indices of beta cell function.

**Glucose Clamp-Based Indices**	
Acute insulin response (AIR)	Area under the curve (AUC) of plasma insulin during first 10 min of hyperglycemic clamp (200 mg/dL)
AIRmax	AIR with arginine stimulation
	Reflects maximal insulin secretion
Disposition index (DI)	Insulin secretion (AIR) adjusted for insulin sensitivity (M value)
**Indices Based on 75 g Oral Glucose Tolerance Test (OGTT)**	
Insulinogenic index (II)	Increment of insulin divided by increment of glucose during first 30 min of 75 g OGTT
AUCinsulin/AUCglucose	AUC of insulin divided by AUC of glucose
Oral DI	Homeostasis model assessment of insulin resistance (HOMA-IR) or Matsuda index is used as insulin sensitivity index; i.e., oral DI is calculated as II/HOMA-IR
**Indices Based on Single Blood Sample**	
HOMA-beta	360 × fasting insulin (mU/L)/(fasting glucose (mg/dL) − 63)
C-peptide to glucose ratio (CPRI)	C-peptide (ng/mL)/glucose (mg/dL) (× 100)
	Assessed in fasting and postprandial states
